# Analysis of genetic diversity and phylogenetic evolution of cattle population in the Ili River Valley region, Xinjiang, based on mtDNA D-loop sequence

**DOI:** 10.1371/journal.pone.0359629

**Published:** 2026-10-23

**Authors:** Guzalnur Amat, Adiljan Kader, Zhenghong Zhan, Yong Tuo, Ziting Wang, Zhijun Zhang, Xiaoyan Wen, Ziyi Ren, Akida Tursun, Ablat Sulayman, Tongjun Guo

**Affiliations:** 1 Xinjiang Key Laboratory of Feed Biotechnology, Urumqi, China; 2 Institute of Feed Research, Xinjiang Academy of Animal Science, Urumqi, China; 3 College of Life Science, Xinjiang Agricultural University, Urumqi, China; 4 Institute of Animal Sciences, Xinjiang Academy of Animal Sciences, Urumqi, China; National Cheng Kung University, TAIWAN

## Abstract

This study used mitochondrial DNA (mtDNA) D-loop region sequence polymorphisms as molecular markers to investigate the genetic structure and maternal origin of six cattle breeds in the Ili River Valley region of Xinjiang, China. Blood samples were collected from six cattle breeds in the region, including Xinjiang Brown cattle (n = 30), Kazakh cattle (n = 33), Altay White-headed cattle (n = 33), Chinese Simmental cattle (n = 34), Chinese Holstein cattle (n = 31), and Angus cattle (n = 22), for DNA extraction. Complete mtDNA D-loop region sequences were obtained from 183 individuals. Sequence alignment, correction and SNP screening were implemented to characterize sequence variation and base composition. Genetic diversity parameters and pairwise genetic distances were estimated, and a neighbor-joining (NJ) phylogenetic tree was constructed. The complete sequences of the mtDNA D-loop region in the six cattle breed populations were 910–911 bp. The average contents of A, G, T, and C were 32.76%, 13.83%, 28.79%, and 24.62%, respectively, and the overall AT content was higher than GC content. A total of 147 SNPs were detected, accounting for 16.10% of the total sequence length. In total, 107 haplotypes were identified, of which 92 were classified as *Bos taurus* and 15 as *Bos indicus*. The *k*, *Hd*, and *Pi* values were 14.24, 0.99, and 0.02, respectively. The constructed NJ phylogenetic tree revealed two major maternal origins—*Bos taurus* and *Bos indicus*—among the six cattle breeds. The six cattle breeds in the Ili River Valley region exhibit rich genetic diversity and two maternal origins, predominantly influenced by *Bos taurus*. These findings provide a theoretical basis for the conservation and rational use of cattle genetic resources in the region.

## Introduction

Cattle are important domestic animals and play an indispensable role in the global agricultural economy by continuously providing essential products, such as meat and milk, for humans [1]. In China, cattle breed resources are highly diverse, with different breeds exhibiting significant variations in production performance and environmental adaptability. The Ili River Valley region—including areas such as Ili Prefecture, the Altay region, and the Tacheng region—features a unique natural environment and highly variable climatic conditions. These factors have fostered the breeding and development of multiple cattle breeds well adapted to the local environment, such as Xinjiang Brown cattle, Kazakh cattle, Chinese Simmental cattle, Altay White-headed cattle, Chinese Holstein cattle, and Angus cattle. These breeds form the core foundation of the local animal husbandry industry and represent extremely valuable genetic resources in China [2].

Xinjiang Brown cattle are a dual-purpose dairy-beef breed developed through three-stage hybridization, with Kazakh cow as the female parent and Swiss Brown, Kostroma, or Ala-Tau bull (the latter two also of Swiss Brown origin) as the male parent [3]. The breed is predominantly distributed in northern Xinjiang, China. It exhibits excellent traits, such as stable genetic performance, strong grazing ability, high roughage tolerance, high adaptability, and strong disease resistance [4]. The Kazakh cattle are a multipurpose local yellow cattle breed used for draught, dairy, and beef. They can tolerate both extreme cold and heat, making them well-suited for extensive feeding systems. The Kazakh cattle also demonstrate strong grazing ability, disease resistance, robust constitution, and stable heredity. They serve as the original breed for cultivating new varieties and exploiting heterosis [5]. Altay White-headed cattle have been shaped by multicultural influences and a complex natural ecological environment [6,7]. They are highly favored by herders in Xinjiang for their exceptional cold tolerance, adaptability, and disease resistance [7]. As a native breed with a long history, Altay White-headed cattle are also valued for their productivity, esthetic appearance, and gentle nature [7]. The Chinese Simmental cattle is a high-quality dual-purpose breed for both milk and meat production in China. It possesses several excellent characteristics, including good milk production and meat yield, strong adaptability, rapid growth rate, superior meat quality, and high slaughter rate [8]. It is the primary breed promoted for beef production in Xinjiang and is used to improve local yellow cattle in rural farming areas. Chinese Simmental cattle hold a significant position in beef and dairy markets due to their excellent milk production performance and carcass quality [9]. In addition, Chinese Simmental cattle exhibit longer lifespans compared to specialized dairy breeds like Chinese Holstein, attributed to their greater rusticity and improved resilience to diseases, disorders, and stressors [10]. The Angus cattle is a small-sized beef breed originating from Scotland. Because of its fresh and tender meat, rich nutritional content, and good taste, it is recognized globally as a typical specialized beef cattle breed and is considered one of the best in terms of meat quality. The meat yield of adult Angus carcasses exceeds 75%. Because of its characteristics, ease of reproduction, rapid growth, early maturity, and strong adaptability, Angus has become the only beef cattle breed in the world whose name is used as a commercial beef brand [11,12]. Chinese Holstein cattle are derived from grading crossbreeding and selection between the local yellow cattle and Holstein, a breed that was mostly imported from Canada, the United States, France, and northern Europe and renamed by the Chinese Ministry of Agriculture in 1992 [13].

Domestic cattle worldwide are generally derived from two main ancestral matrilines: *Bos taurus* (taurine cattle) and *Bos indicus* (zebu cattle) [14]. Chinese cattle exhibit a typical dual maternal origin structure, and previous studies have shown that northern Chinese cattle are predominantly influenced by *Bos taurus*, while southern cattle are more affected by *Bos indicus* [15]. However, the matrilineal composition and divergence pattern of cattle in the Ili River Valley remain unclear.

Mitochondrial DNA (mtDNA) is a stable, maternally inherited molecular marker that does not undergo recombination, making it powerful for tracing maternal lineages, matriline genetic diversity, and phylogenetic origin in livestock [16]. As mtDNA only reflects matrilineal inheritance rather than whole-genomic admixture or genetic dilution, clarifying the matriline diversity and phylogenetic relationships is crucial for their scientific conservation and sustainable use [17,18]. The D-loop is a noncoding control region in mtDNA, functioning both as a regulatory element and a key molecular marker characterized by high variability and neutral base mutations. Recent bioinformatics analyses across multiple species have confirmed that the mtDNA D-loop region is among the most conservative and stable sequences in the mitochondrial genome. It directly reflects genetic diversity, population genetic structure, and subspecies differentiation [19]. The D-loop region has been widely used in studies of genetic diversity and phylogenetic differentiation in domestic animals. For example, studies on goats [20,21], sheep [22,23], horses [24–26], donkeys [27,28], camels [29], pigs [30,31], chickens [32], and yaks [33–35] have successfully used in-depth analyses of the mtDNA D-loop region to elucidate genetic relationships and evolutionary origins among different breeds.

The cattle breeds in the Ili River Valley region have each developed distinct genetic characteristics through a long history of natural selection and artificial breeding. However, the rapid development of modern animal husbandry has posed significant challenges to the maternal genetic structure of local breeds. Frequent crossbreeding has shaped complex maternal genetic backgrounds in local populations, highlighting the need to clarify matriline composition and genetic relationships. Therefore, an in-depth investigation of maternal genetic diversity and matriline phylogenetic structure based on mtDNA is crucial for their scientific conservation and sustainable utilization [36,37].

Currently, comprehensive studies on the maternal genetic diversity and matriline structure of the mtDNA D-loop region in six cattle breeds from the Ili River Valley—Xinjiang Brown cattle, Kazakh cattle, Chinese Simmental cattle, Altay White-headed cattle, Chinese Holstein cattle, and Angus cattle—are relatively lacking. This study aimed to conduct a comprehensive sequence analysis of the mtDNA D-loop regions of these six breeds to accurately assess their maternal genetic diversity, matriline structure, and phylogenetic relationships.

## Materials and methods

### Ethics statement

All procedures related to cattle sample collection and genetic research in this study were approved by the Animal Welfare and Ethics Committee of Xinjiang Agricultural University, Urumqi, China (Approval Number: 2024045). All animal handling and sample collection were performed in strict accordance with the National Animal Welfare Guidelines of China to ensure animal welfare and minimize animal suffering.

### Sample collection and DNA extraction

To ensure sample representativeness, field investigations were conducted in breeding areas across various counties and cities in the Ili River Valley region. The herdsmen were interviewed about the breeding histories of the cattle breeds, and the relevant pedigree records were reviewed. A total of 183 tail vein blood samples were collected from six dominant cattle breeds: Xinjiang Brown cattle (n = 30), Kazakh cattle (n = 33), Altay White-headed cattle (n = 33), Chinese Simmental cattle (n = 34), Chinese Holstein cattle (n = 31), and Angus cattle (n = 22). Samples were obtained from 15 breeding sites across 11 counties within the Ili Prefecture, Altay region, and Tacheng region. Blood samples were transported to the laboratory under low-temperature conditions and stored at −20°C for use. Fig 1 shows the geographical distribution of the sampling locations for each population.

**Fig 1:**
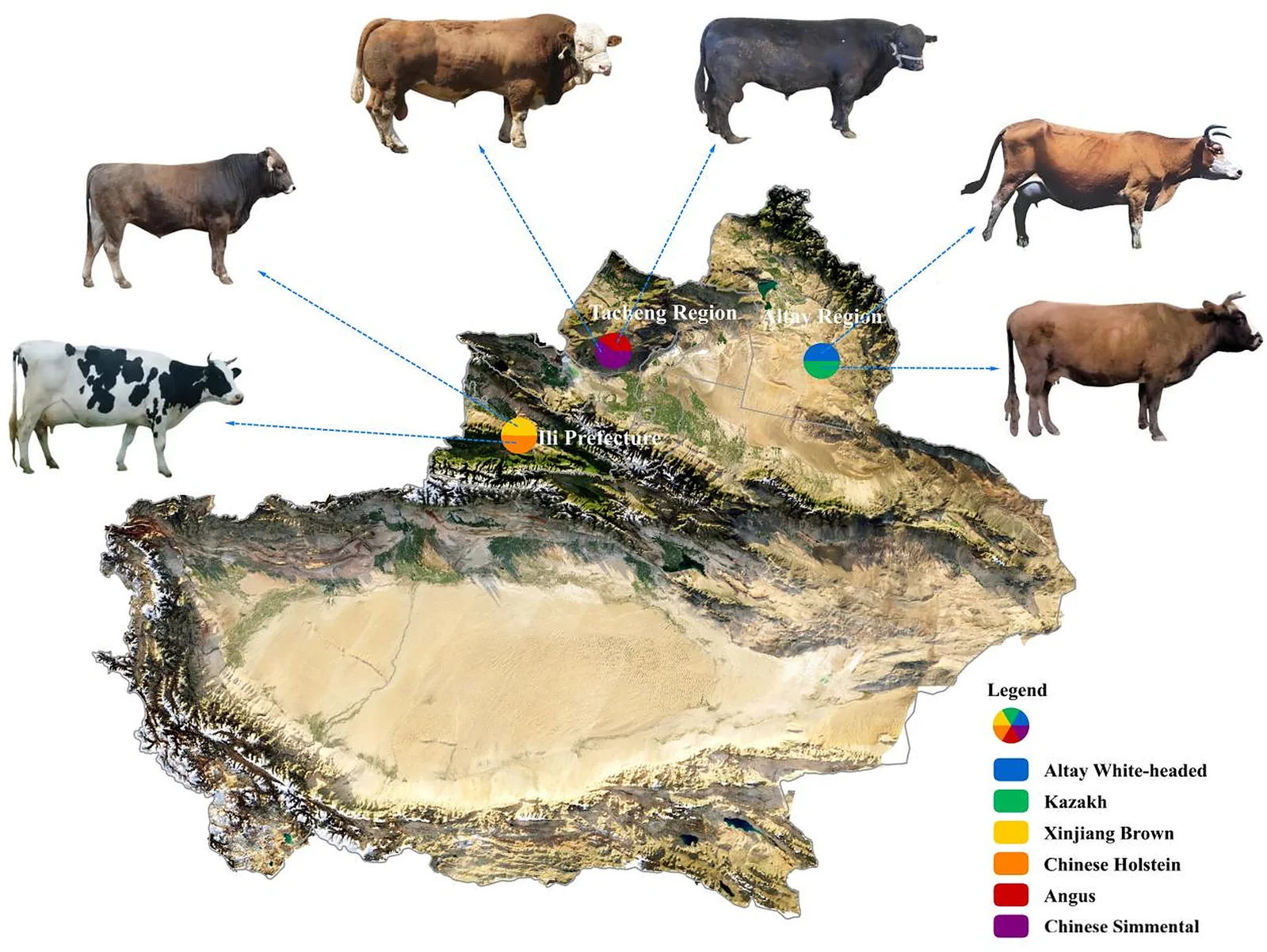
Geographical distribution of the sampling locations for the six cattle breeds in the Ili River Valley region. The public-domain data used in this figure were obtained from Natural Earth, a public domain map dataset available for free use in any type of project.

Using the complete mtDNA D-loop sequence of European cattle determined by Anderson *et al*. [38] (GenBank accession number: V00654) as the reference, and sequences of American Brahman cattle (GenBank accession number: DQ887761) and Indian Nellore cattle (GenBank accession number: AY126697) as external control sequences, we assessed genetic variation among the six local cattle breeds. The three external reference and control sequences were excluded from this sample count and not incorporated into population genetic analyses.

### Genomic DNA extraction and detection

Genomic DNA was extracted using a tissue DNA extraction kit (Tiangen Biochemical Technology, Beijing) following the manufacturer’s instructions. This kit supports DNA extraction from blood specimens as specified by the supplier. DNA quality and concentration were assessed using 1.5% agarose gel electrophoresis and ultraviolet spectrophotometry (Nanodrop 2000, Thermo Fisher Scientific, USA). The qualified genomic DNA concentrations ranged from 14–42 nmol/μL, the DNA concentration measured by Nanodrop was used to calculate the template volume for subsequent PCR amplification. The DNA samples were then stored at −20°C for later use.

### Polymerase chain reaction amplification and sequencing

Based on the published bovine mtDNA D-loop region sequence (GenBank accession number: AF492351.1), upstream and downstream primers were designed using Premier 5 software. The primer sequences were: Forward: 5’-AACTGCAGTCTCACCATCAAC-3’ and Reverse: 5’-GTTAACAGGAAGGCTGGGACC-3’. This primer pair amplified a 1063-bp segment of the bovine mtDNA D-loop. PCR was performed in a 50 μL volume reaction mixture containing 25 μL 2 × Taq PCR MasterMix, 2 μL of each forward and reverse primer (10 μmol/L), 2 μL template DNA, and 19 μL nuclease-free water, under the following conditions: initial denaturation at 95°C for 5 min; 32 cycles of denaturation at 94°C for 30 s, annealing at 57°C for 30 s, and extension at 72°C for 30 s; followed by a final extension at 72°C for 10 min. The reaction products were stored at 4°C. The PCR products were electrophoresed on 1.5% agarose gels, purified using a gel recovery kit, and sent for sequencing at Shanghai Shenggong Bioengineering Co., Ltd.

### Data analysis

Using the D-loop sequence of European cattle mtDNA determined by Anderson *et al* [38]. (GenBank accession number: V00654) as the reference, the complete sequences were aligned and corrected using DNAMAN, followed by homologous sequence alignment using the Clustal-W program [39] for sequence comparison [40]. Polymorphic sites and corresponding haplotypes were identified using Mega 6.0 software [41]. Single-nucleotide polymorphism (SNP) sites, the number of haplotypes (*H*), haplotype diversity (*Hd*), nucleotide diversity (*Pi*), and the average number of nucleotide differences (*K*) were calculated using DnaSPv5.10 software [42]. A neighbor-joining (NJ) phylogenic tree was constructed using MEGA 6.0 with 1000 bootstrap replicates [41]. Additionally, a haplotype network was constructed using Network 5.0.1.1 software [43] to illustrate the relationships among haplotype sequences.

## Results

### PCR amplification of the mtDNA D-loop region

The amplification conditions of the designed primers for the cattle mtDNA D-loop region were optimized, resulting in successful amplification for most of the samples. The PCR products were assessed using 1.5% agarose gel electrophoresis, showing a single, bright band approximately 1063 bp in size (S1 Fig). The figure shows that the PCR products had high purity and concentration, confirming successful amplification and suitability for subsequent analysis.

### Genetic polymorphism in the mtDNA D-loop region

The full-length sequence of the mtDNA D-loop region from 183 individuals across six cattle breeds in the Ili River Valley region was 910–911 bp. A total of 147 variable sites were identified, comprising 58 singleton variable sites (all biallelic) and 89 parsimony-informative sites. Among these parsimony-informative sites, 56 were biallelic, 2 were triallelic, and no quadrallelic sites were detected. Across the six breeds, 107 distinct haplotypes were detected. The average number of nucleotide differences (*k*) was 14.24, haplotype diversity (*Hd*) was 0.99, and nucleotide diversity (*Pi*) was 0.02. Five types of mutations were observed in the D-loop region, transitions, transversions, insertions, deletions, and sites with both transitions and transversions. The nucleotide composition across all haplotypes for A, T, C, G, A + T, and G + C were 32.76%, 28.79%, 24.62%, 13.83%, 61.55%, and 38.45%, respectively (S1 Table). The A + T content was substantially more common than the G + C content. The 147 variable sites accounted for approximately 16.10% of the total sequence length (S2 Fig).

### Haplotypes and sequence polymorphisms

The haplotypes of the European cattle sequence determined by Anderson *et al.* [38] and the D-loop sequences of the Brahman and Nellore cattle were designated as H1, H2, and H3, respectively. A total of 107 haplotypes were identified among 183 individuals, denoted H1–H107 (S2 Fig). Among these haplotypes, the reference haplotype H1 was found in two individuals from the Altay White-headed cattle and three individuals from the Chinese Holstein cattle. Haplotype H2, which is specific to American Brahman cattle, was also detected in several individuals introduced to China. H2 closely resembles H1, differing by only two substitutions: a G → A transition at position 556 and an A → G transition at position 717. H2 clusters with *Bos taurus* reference haplotype H1. Haplotype H3 differs significantly from both H1 and H2, with a relatively high number of nucleotide differences. Haplotypes associated with zebu cattle were common in the Ili River Valley population, accounting for 16.98% of the 107 haplotypes identified across the six cattle breeds. Among the six cattle breeds in the Ili River Valley region, Angus cattle exhibited the highest proportion of *Bos indicus* lineage (40.91%), followed by Chinese Holstein cattle (16.20%) and Chinese Simmental cattle (11.76%). Based on sequence differences among the 107 haplotypes, 15 haplotypes, H93–H107 were inferred to be specific to *Bos indicus*, whereas the remaining 92 haplotypes were attributed to *Bos taurus*. As shown in S2 Fig, there are 10 nucleotide sites among the 183 sequences that stably distinguish *Bos indicus* haplotypes from *Bos taurus* haplotypes, all of which involved base transitions. The specific base variations distinguishing *Bos indicus* and *Bos taurus* haplotypes are shown in Table 1.

**Table 1:** Base variations in *Bos indicus* and *Bos taurus* haplotypes.

No.	Position	*Bos indicus* → *Bos taurus*
**1**	168	A → G
**2**	291	A → G
**3**	311	A → G
**4**	325	C → T
**5**	339	C → T
**6**	346	C → T
**7**	405	A → G
**8**	754	C → T
**9**	782	C → T
**10**	845	C → T

Position 1 corresponds to position 15792 in GenBank accession number V00654.

The number of haplotypes identified per breed was 15–26. Altay White-headed cattle and Kazakh cattle exhibited the least haplotypes, whereas Xinjiang Brown cattle, Angus cattle, Chinese Holstein cattle, and Chinese Simmental cattle had the most (Table 2). Among the 107 haplotypes, 94 were breed-specific and 13 were shared either among or within breeds. Of the 13 shared haplotypes, Hap9 and Hap10 had relatively high frequencies of 6.56% and 4.92%, respectively, corresponding to 12 and 9 individuals. The remaining haplotypes were represented by 2–5 individuals (Table 2).

**Table 2:** Haplotype distribution among the six cattle breeds.

Breeds	*Bos taurus* haplotypes	*Bos indicus* haplotypes	Haplotype number	Proportion of *Bos indicus* bloodline (%)
**AWH**	**2H1**, 2H4, H5, **H6**, 2H7, **H8**, **7H9**, **6H10**, 2H11, 2H12, H13, H14, 2H15	H94, 2H95	15	9.09
**KAZ**	**2H10**, 6H16, 3H17, 2H18, **2H19**, H20, **H21**, H22, 2H23, **2H24**, 2H25, 2H26, 3H27, 2H28	2H93	15	6.06
**XB**	**4H9**, **H10**, **2H19**, 2H29, H30, H31, **H32**, H33, H34, H35, H36, H37, H38, H39, 2H40, H41, 2H42, 2H43, H44, H45, 2H46	–	21	0.00
**ANG**	H47, **H48**, H49, H50, H51, H52, H53, H54, H55, H56, H57, **H58**, H59	**H3**, H96, H97, H98, H99, **H100**, H101, H102, H103	22	40.91
**HOL**	**3H1**, **2H8**, **H9**, **H32**, **H48**, **H58**, 2H60, H61, H62, H63, 2H64, H65, 2H66, H67, H68, H69, 2H70, H71, H72	**H100**, H104, H105, 2H107	23	16.13
**SIM**	**2H6**, **H8**, **H21**, **H24**, H73, 2H74, H75, H76, H77, H78, 3H79, H80, H81, H82, H83, H84, H85, H86, 2H87, 2H88, H89, H90, H91, H92	**3H3**, H106	26	11.76

Breed codes: AWH, Altay White-headed cattle; KAZ, Kazakh cattle; XB, Xinjiang Brown cattle; ANG, Angus cattle; HOL, Chinese Holstein cattle, and SIM, Chinese Simmental cattle. The numbers preceding each haplotype indicate the number of individuals in which the haplotype was detected. Haplotypes in bold are shared among breeds; others are breed-specific.

### Genetic distances among breeds and phylogenetic analysis

The Kimura two-parameter genetic distances among the six breeds ranged from 0.01 (between Altay White-headed cattle and Xinjiang Brown cattle) to 0.03 (between Angus cattle and Chinese Simmental cattle) (Table 3). The largest genetic distance was observed between Angus and Chinese Simmental cattle (0.03), followed by the distances between Angus and Chinese Holstein cattle (0.03) and between Kazakh and Angus cattle (0.03). The smallest genetic distance was between Altay White-headed and Xinjiang Brown cattle (0.01) (Table 3). The NJ phylogenetic tree of the 107 haplotypes of the six cattle breeds is shown in Fig 2. It shows that the haplotypes clustered into two major groups: Types I and II. Type I included all 91 *Bos taurus* haplotypes, and Type II included 16 haplotypes specific to *Bos indicus*. These 16 haplotypes correspond to those showing the characteristic sequence features of *Bos indicus* cattle, as shown in S2 Fig.

**Table 3:** Genetic distances among the six cattle breeds.

Breeds	AWH	KAZ	XB	ANG	HOL	SIM
**AWH**						
**KAZ**	0.01					
**XB**	0.01	0.01				
**ANG**	0.03	0.03	0.03			
**HOL**	0.02	0.02	0.01	0.03		
**SIM**	0.01	0.02	0.01	0.03	0.02	

Breed codes: AWH, Altay White-headed cattle; KAZ, Kazakh cattle; XB, Xinjiang Brown cattle; ANG, Angus cattle; HOL, Chinese Holstein cattle; SIM, Chinese Simmental cattle.

**Fig 2:**
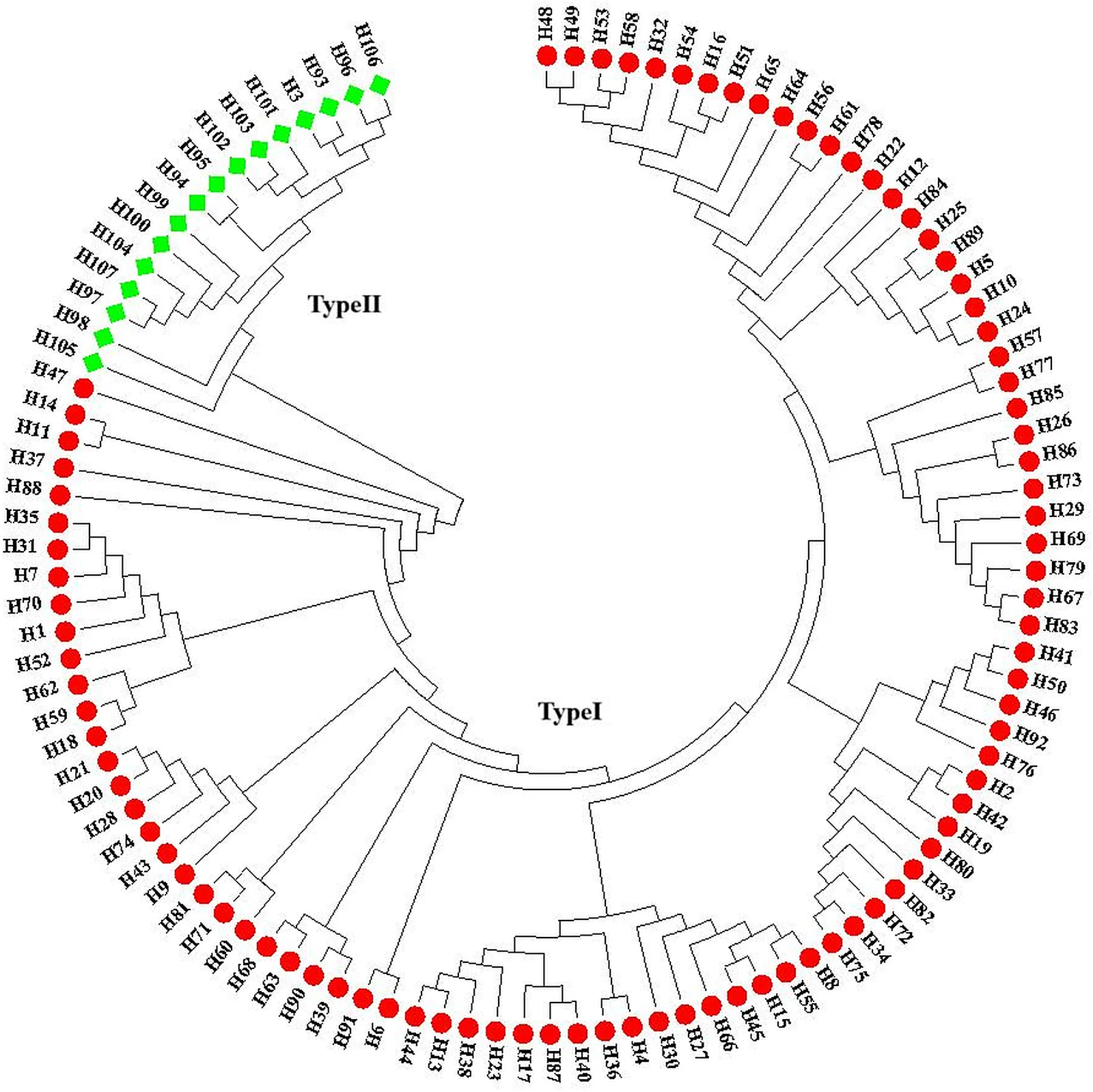
Neighbor-joining tree based on the Kimura two-parameter distances for 107 haplotypes. Type I (red solid circle) represents *Bos taurus*; Type II (green diamond) represents *Bos indicus*.

### Network analysis of haplotypes

To further explore the relationships among the six cattle breeds, network diagrams were constructed using the median-joining method in Network software based on the sequence data of 107 haplotypes (Fig 3). The resulting evolutionary network clearly separated the haplotypes into two major groups: *Bos taurus* (Type I, left) and *Bos indicus* (Type II, right). Within the *Bos taurus* group, haplotypes H_1, H_8, and H_79 exhibited the highest frequencies and occupied prominent central positions. The *Bos taurus* haplotypes were scattered, forming a large and complex cluster. The *Bos indicus* haplotypes showed varying degrees of genetic relationships with the H_3 haplotype in the Indian zebu cattle. Notably, haplotype H_101 exhibited the highest frequency among *Bos indicus* haplotypes and held a central position in the Type II network, with other haplotypes radiating from it.

**Fig 3:**
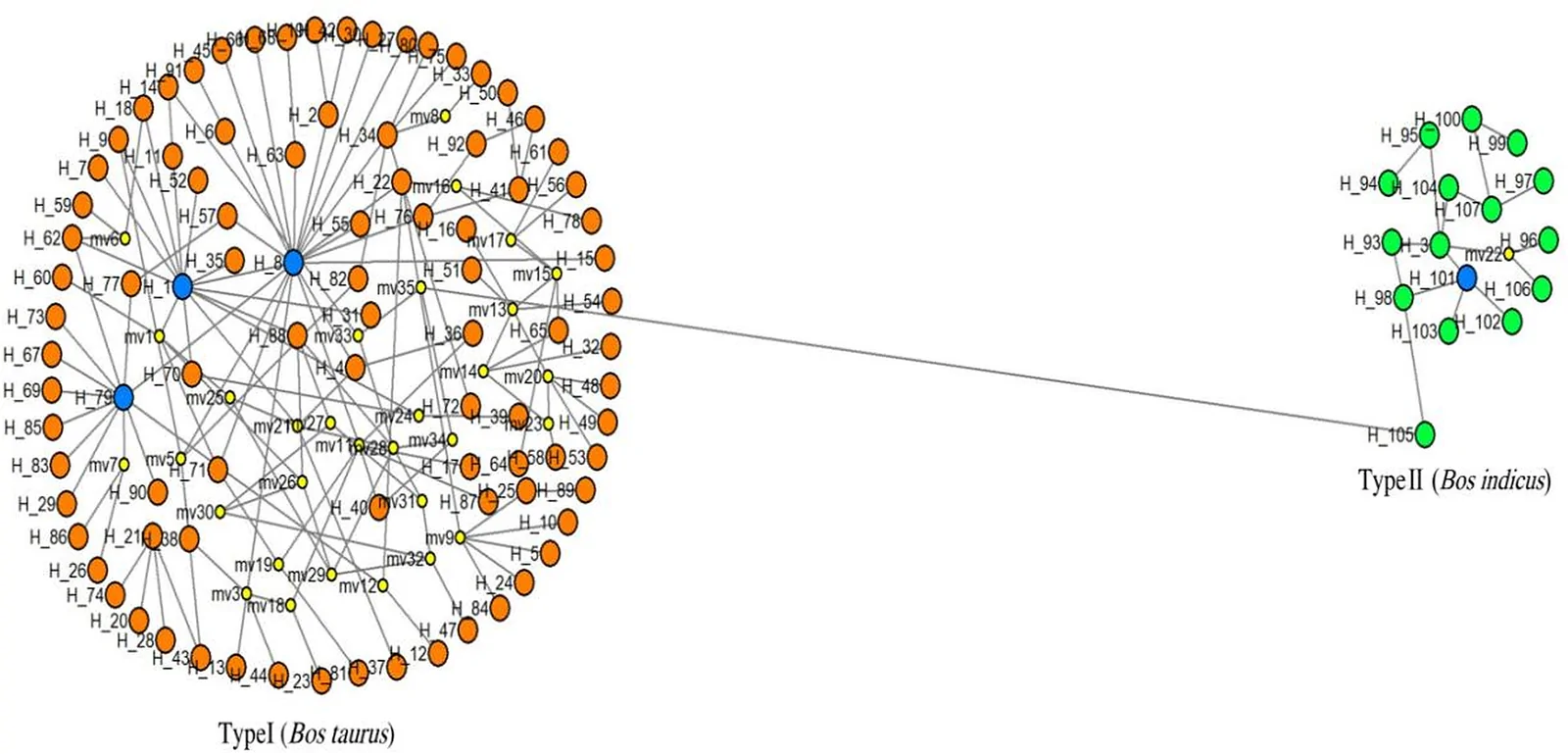
Reduced median-joining networks of mtDNA D-loop haplotypes among the six cattle breeds. Large circles represent haplotypes; small circles represent median vectors.

## Discussion

Wang *et al*. [44], Bai *et al*. [45], Liu *et al*. [46], Chen *et al*. [47], and Hikmawaty *et al*. [48] analyzed the nucleotide composition of the mtDNA D-loop region in Kazakh, Qinchuan, Southern Anhui, Mongolian, and Bali Yellow cattle, respectively, reporting A + T contents of 58.40%–63.40%. In the present study, analysis of the mtDNA D-loop region in 183 individuals revealed an A + T content of 61.55%, which was significantly higher than the C + G content of 38.45%. These findings are consistent with those of previous studies and suggest that the complete mtDNA genome and the D-loop region share similar base compositions in *Bos taurus*. This conforms to the base composition characteristics of vertebrate mtDNA and the general base composition patterns of mammalian mtDNA [49,50]. The A + T content was substantially higher than the G + C content, indicating an AT bias. The high proportion of variable sites (16.10% of the total sequence length) indicates a high level of genetic variation among cattle breeds sampled in the Ili River Valley.

Transitions and transversions are two types of nucleotide substitutions that serve as important parameters for assessing DNA sequence evolution. Transitions, which occur between structurally similar bases, are generally more frequent than transversions. However, transitions occur more frequently between species with close genetic relationships. As genetic divergence increases, transitions tend to reach saturation, and the ratio of transitions to transversions decreases with longer evolutionary distances. Similarly, during mitochondrial genome evolution, transitions occur at much higher frequencies than transversions [51]. The raw amplified D-loop fragment was 945 bp in length. After trimming primer sequences and low-quality regions, the valid sequence used for subsequent analyses ranged from 910 to 911 bp. Analysis of the 183 sequences revealed 147 variable sites, which were predominantly concentrated at both ends of the sequence. This suggests that the terminal regions of the D-loop may constitute the primary hypervariable segments. Four types of variations were identified: transitions, transversions, deletions, and insertions. Among these, transitions were the most prevalent, consistent with the findings of Wang *et al*. [52] and Sun *et al*. [53]. Some variable sites exhibited both transversion and transition events. A/T transversions accounted for 53.57% of all transversions, aligning with the findings by Zhang *et al*. [35] regarding Tibetan yaks, which also showed a predominance of A/T transversions. It is speculated that the relatively high mutation rate in the mtDNA D-loop region among cattle populations in the Ili River Valley may be influenced by the region’s unique environmental conditions.

The study of genetic diversity in livestock is essential for understanding their unique genetic characteristics and plays a crucial role in the objective assessment, exploration, conservation, development, and utilization of genetic resources. The mtDNA D-loop region is rich in A + T bases and is recognized as a hypermutable segment of the genome [54]. Genetic diversity is a key component of species evolution. Species with high genetic diversity tend to produce offspring with stronger adaptability and are more likely to expand, whereas low genetic diversity can reduce a population’s adaptability [55]. Nucleotide diversity in mtDNA is an important indicator for assessing population genetic differentiation and the degree of population polymorphism, whereas haplotype diversity reflects the degree of variation within a population. Higher values for both indices indicate greater genetic diversity [56]. The diversity of animal genetic resources provides a foundation for breed selection and genetic improvement. Populations with richer genetic diversity offer more breeding material and thus greater genetic potential for selection [57,58]. In this study, we conducted a maternal genetic analysis of 183 individuals from six cattle breeds in the Ili River Valley region based on mtDNA D-loop sequences. A total of 107 haplotypes were identified. Specifically, Altay White-headed cattle, Kazakh cattle, Xinjiang Brown cattle, Angus cattle, Chinese Holstein cattle, and Chinese Simmental cattle had 10, 11, 17, 18, 16, and 21 specific haplotypes, respectively, indicating that each breed harbors unique maternal genetic information. Additionally, 164 individuals of *Bos taurus* origin (164/183, 89.62%) shared 92 haplotypes, indicating high genetic diversity. In contrast, 19 individuals of *Bos indicus* origin (19/183, 10.38%) shared only 15 haplotypes, suggesting relatively lower genetic diversity. Among the six cattle breeds, only the Xinjiang Brown cattle lacked the abovementioned *Bos indicus* haplotypes. Despite this, Xinjiang Brown cattle had 21 *Bos taurus* haplotypes, reflecting a high level of genetic diversity. Moreover, except for the Xinjiang Brown cattle, the other five breeds had both *Bos taurus* and *Bos indicus* haplotypes, indicating the presence of gene flow among them. Hap9 and Hap10 occurred at relatively high frequencies among the shared haplotypes, indicating that these two haplotypes were the predominant haplotypes in the sampled populations.

Haplotype diversity (*Hd*) and nucleotide diversity (*Pi*) are the two primary indicators used to evaluate mtDNA genetic diversity, and both correlate positively with overall genetic diversity. In this study, analysis of the mtDNA sequences from 183 individuals revealed a haplotype diversity (*Hd*) of 0.99 and a nucleotide diversity (*Pi*) of 0.02. These results indicate that the six cattle breeds in the Ili River Valley region exhibit relatively high maternal genetic diversity, which may be associated with the region’s unique geography. Wang *et al*. [44] analyzed the mitochondrial genomes of 179 individuals from seven Kazakh cattle populations in the Xinjiang region and reported an *Hd* value of 0.97 and a *Pi* value of 0.01. Chen *et al*. [47] found that the *Hd* and *Pi* values for Mongolian cattle were 1.00 and 0.01, respectively. Zhang *et al*. [35] reported *Hd* and *Pi* values of 0.98 and 0.01, respectively, for the mtDNA D-loop region of Tibetan yaks. Lai *et al*. [59] sequenced the D-loop region of five yellow cattle breeds (or groups) in Sichuan, China, and observed an *Hd* value of 0.5–1.0 and a *Pi* value of 0.00–0.29. These results are broadly consistent with those of the present study. The six cattle breeds analyzed in this study were mainly distributed in the Ili River Valley region. The region’s abundant pastures and vast geography facilitate gene flow among cattle populations, contributing to their high genetic diversity.

Genetic distance is a key indicator for assessing genetic relationships between populations. Smaller distances indicate closer genetic relationships, whereas larger distances reflect greater divergence. Among the six cattle breeds studied, genetic distances ranged from 0.01 to 0.03. The relatively low genetic distance of the Xinjiang Brown cattle is attributable to its exclusive *Bos taurus* lineage. The smallest genetic distance was observed between the Altay White-headed and Xinjiang Brown cattle (0.01), suggesting a close genetic relationship. Altay White-headed, Kazakh, Xinjiang Brown, Angus, and Chinese Holstein cattle showed relatively high average intra-population genetic distances due to the presence of both *Bos taurus* and *Bos indicus* lineages. This supports the observation by Lei *et al*. [60] that Chinese Yellow cattle exhibit greater D-loop sequence divergence than European, Indian zebu, and African cattle. In contrast, studies by Bradley *et al*. [61] on European and African cattle and Mannen *et al*. [62] on Japanese Black cattle reported lower D-loop sequence divergence, likely due to their pure European *Bos taurus* lineage.

In this study, phylogenetic trees were constructed and network analyses were performed based on 107 haplotypes from the mtDNA D-loop region sequences of six cattle breeds. The clustering analysis revealed that these breeds clustered into two major groups: *Bos taurus* and *Bos indicus*. Although all six breeds exhibited evidence of dual maternal origins, the degree of influence varied. The network topology shows distinct grouping of *Bos taurus* and *Bos indicus* lineages, which indicates that the Chinese Yellow cattle may have a mixed origin from *Bos indicus* and *Bos taurus*. Overall, the maternal lineage composition was biased toward *Bos taurus*. In a study of Chinese Yellow cattle, Chen *et al*. [63] found that northern populations were predominantly influenced by *Bos taurus*, whereas southern populations were predominantly influenced by *Bos indicus*. Similarly, Liu *et al*. [64] and Lei *et al*. [60] confirmed through D-loop sequence analysis the dual maternal origins of the Chinese Yellow cattle—*Bos taurus* and *Bos indicus*. Among the six cattle breeds in the Ili River Valley region, the *Bos taurus* clade comprised 92 haplotypes, accounting for 85.98% (92/107) of the total, indicating relatively high maternal genetic diversity. In contrast, the *Bos indicus* clade contained only 15 haplotypes, accounting for 14.02% (15/107) of the total, suggesting relatively lower maternal genetic diversity. These results suggest that *Bos taurus* has had a greater maternal influence on the genetic makeup of these populations. Consistent with this finding, other studies [46,53,65–69] have reported that southern Chinese Yellow cattle are more influenced by *Bos indicus*, whereas northern populations are more influenced by *Bos taurus*, likely reflecting regional differentiation. The finding in this study that the genetic diversity of *Bos indicus* is lower than that of *Bos taurus*. This discrepancy may be attributed to limited haplotype variation among the introduced *Bos indicus* ancestors in Xinjiang, the predominance of a single lineage, relatively few accumulated mutations, and relatively recent introduction of *Bos indicus* into the region. This study is based on maternally inherited mtDNA D-loop sequences, which can effectively reveal matriline genetic diversity and phylogenetic origin. However, mtDNA only reflects maternal inheritance and cannot fully represent genome-wide introgression or overall genetic dilution. Future studies using nuclear genome markers will help to comprehensively evaluate the genetic structure and admixture history of these cattle populations.

## Conclusions

In this study, analyses of base composition, genetic diversity, and NJ phylogenetic relationships were performed using the mtDNA D-loop region sequences of 183 individuals representing six cattle breeds in the Ili River Valley region. The *Hd* was 0.99 and *Pi* was 0.02, indicating substantial maternal genetic diversity across the six breeds. Phylogenetic tree and evolutionary network analyses revealed that of the 107 haplotypes identified, 92 (representing 164 individuals or 85.98%) clustered within the *Bos taurus* clade, whereas only 15 (representing 19 individuals or 14.02%) clustered within the *Bos indicus* clade. These results indicate the presence of two major maternal lineages—*Bos indicus* and *Bos taurus*—with a clear predominance of *Bos taurus* among the cattle populations in this region. These findings provide valuable insights into the maternal genetic structure of cattle in the Ili River Valley region and have important implications for ecological breed distribution, selection programs, and conservation of genetic resources.

## Supporting information

S1 Fig Agarose gel electrophoresis of mtDNA D-loop PCR products. (DOCX)

S2 Fig Polymorphic sites of 107 mtDNA D-loop haplotypes identified in six cattle breeds. (DOCX)

S1 Table Base pair composition of mtDNA D-loop of six cattle breeds. (DOCX)

S1 Raw Images Original uncropped and unadjusted gel image for PCR product electrophoresis presented in S1 Fig. (PDF)
