# Supplementary material for: Analysis of genetic diversity and phylogenetic evolution of cattle population in the Ili River Valley region, Xinjiang, based on mtDNA D-loop sequence
Source: PLoS One. 2026 Oct 23;21(10):e0359629. doi: 10.1371/journal.pone.0359629 (PMC13693866; doi:10.1371/journal.pone.0359629)
Supplement: Supplementary file 1 [file pone.0359629.s004.pdf]

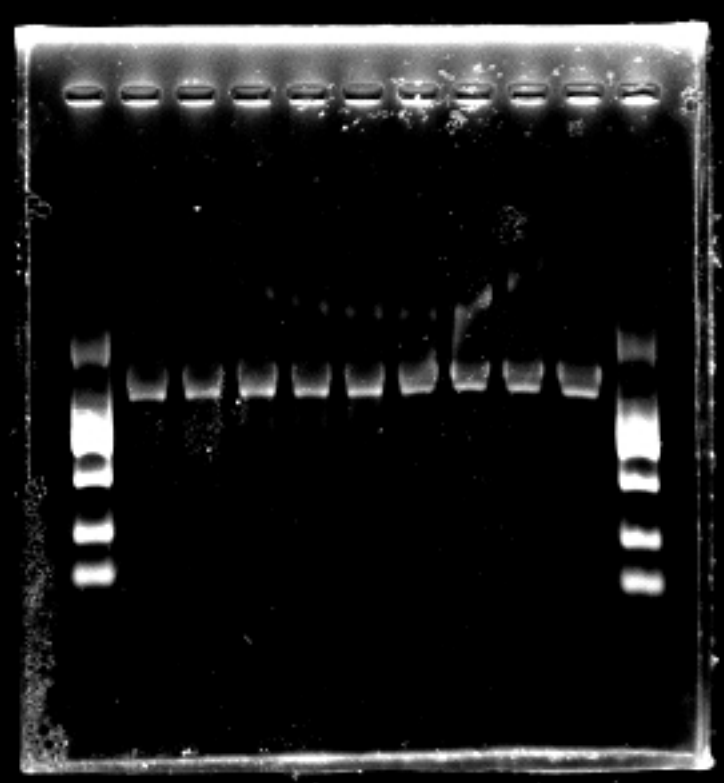

Loading order:
Lanes M (left and right): DL2000 DNA Marker
Lanes 1–9: mtDNA D-loop PCR products
Image capture: Gel-Doc gel imaging system
Corresponding figure in manuscript: S1 Fig
No unused lanes on this gel
