# Supplementary material for: Analysis of genetic diversity and phylogenetic evolution of cattle population in the Ili River Valley region, Xinjiang, based on mtDNA D-loop sequence
Source: PLoS One. 2026 Oct 23;21(10):e0359629. doi: 10.1371/journal.pone.0359629 (PMC13693866; doi:10.1371/journal.pone.0359629)
Supplement: Supplementary file 3 [file pone.0359629.s002.docx]

Continued from S2 Fig

**S2 Fig. Polymorphic sites of 107 mtDNA D-loop haplotypes identified in six cattle breeds. (·indicates the same base).**
