# Supplementary material for: Analysis of genetic diversity and phylogenetic evolution of cattle population in the Ili River Valley region, Xinjiang, based on mtDNA D-loop sequence
Source: PLoS One. 2026 Oct 23;21(10):e0359629. doi: 10.1371/journal.pone.0359629 (PMC13693866; doi:10.1371/journal.pone.0359629)
Supplement: Supplementary file 4 [file pone.0359629.s003.docx]

**S1 Table. Base pair composition of mtDNA D-loop of six cattle breeds.**

| **Population** | **N** | **A**（%） | **T**（%） | **C**（%） | **G**（%） | **A+T**（%） | **G+C**（%） |
| --- | --- | --- | --- | --- | --- | --- | --- |
| **AWH** | 33 | 32.74 | 28.79 | 24.68 | 13.79 | 61.53 | 38.47 |
| **KAZ** | 33 | 32.75 | 28.95 | 24.43 | 13.87 | 61.69 | 38.31 |
| **XB** | 30 | 32.75 | 28.94 | 24.47 | 13.85 | 61.69 | 38.31 |
| **ANG** | 22 | 32.78 | 28.50 | 24.95 | 13.78 | 61.28 | 38.72 |
| **HOL** | 31 | 32.77 | 28.79 | 24.59 | 13.84 | 61.56 | 38.44 |
| **SIM** | 34 | 32.78 | 28.79 | 24.60 | 13.83 | 61.57 | 38.43 |
| **Average** | 183 | 32.76 | 28.79 | 24.62 | 13.83 | 61.55 | 38.45 |

Note: Breed codes: AWH, Altay White-headed cattle; KAZ, Kazakh cattle; XB, Xinjiang Brown cattle; ANG, Angus cattle; HOL, Chinese Holstein cattle; SIM, Chinese Simmental cattle.
