# Supplementary material for: Analysis of genetic diversity and phylogenetic evolution of cattle population in the Ili River Valley region, Xinjiang, based on mtDNA D-loop sequence
Source: PLoS One. 2026 Oct 23;21(10):e0359629. doi: 10.1371/journal.pone.0359629 (PMC13693866; doi:10.1371/journal.pone.0359629)
Supplement: Supplementary file 5 [file pone.0359629.s001.docx]

**S1 Fig. Agarose gel electrophoresis of mtDNA D-loop PCR products.**
